# Cardiac sequelae after COVID-19: Results of a 1-year follow-up study with echocardiography and biomarkers

**DOI:** 10.3389/fcvm.2022.1067943

**Published:** 2022-12-21

**Authors:** Gabriela Matejova, Martin Radvan, Elis Bartecku, Martin Kamenik, Lumir Koc, Jana Horinkova, Lubica Sykorova, Radka Stepanova, Petr Kala

**Affiliations:** ^1^Department of Internal Medicine and Cardiology, University Hospital Brno, Brno, Czechia; ^2^Faculty of Medicine, Masaryk University, Brno, Czechia; ^3^Department of Psychiatry, University Hospital Brno, Brno, Czechia; ^4^Clinic of Pulmonary Disease and Tuberculosis, University Hospital Brno, Brno, Czechia; ^5^Department of Pharmacology, Masaryk University, Brno, Czechia

**Keywords:** COVID-19, SARS-CoV-2, echocardiography, troponin, NT-proBNP, survilence

## Abstract

**Objective:**

To evaluate the need for cardiac monitoring in unselected patients recovered from COVID-19 and to estimate the risk of heart complications after severe acute respiratory syndrome coronavirus 2 (SARS-CoV-2).

**Materials and methods:**

During March 2020 and January 2021, 106 patients who had recovered from SARS-CoV-2 (alpha and beta variants) were enrolled in prospective observational cohort study CoSuBr (Covid Survivals in Brno). The diagnosis was based on a reverse transcription-polymerase chain reaction swab test of the upper respiratory tract. Demographic parameters, patient history, clinical evaluation, cardiac biomarkers, ECG and echocardiography were recorded during three visits (Visit 1 at least 6 weeks after infection, Visit 2 three months later, and Visit 3 one year after Visit 1).

**Results:**

58.5% of the study group (*n* = 106) were female, while the mean age was 46 years (range 18–77 years). The mean time interval between the onset of infection and the follow-up visit was 107 days. One quarter (24.5%) of the patients required hospitalization during the acute phase of the disease; the rest recovered at home. 74% suffered a mild form of the disease, with 4.8, 18.1, and 2.9% suffering moderate, severe, and critical forms, respectively. At the time of enrolment, 64.2% of the patients reported persistent symptoms, while more than half of the whole group (50.9%) mentioned at least one symptom of possible cardiac origin (breathing problems, palpitations, exercise intolerance, fatigue). In the 1-year follow-up after COVID-19 infection, left ventricle ejection fraction showed no significant decrease [median (IQR) change was −1.0 (−6.0; 4.0)%, *p* = 0.150], and there were no changes of troponin (mean change −0.1 ± 1.72 ng/L; *p* = 0.380) or NT-proBNP [median (IQR) change 2.0 (−20.0; 29.0) pg/mL; *p* = 0.315]. There was a mild decrease in right ventricle end diastolic diameter (-mean change 2.3 ± 5.61 mm, *p* < 0.001), while no right ventricle dysfunction was detected. There was very mild progress in left ventricle diastolic diameter [median (IQR) change 1.0 (−1.0; 4.0) mm; *p* = 0.001] between V1 and V3, mild enlargement of the left atrium (mean change 1.2 ± 4.17 mm; *p* = 0.021) and a non-significant trend to impairment of left ventricle diastolic dysfunction. There was a mild change in pulmonary artery systolic pressure [median (IQR) change 3.0 (−2.0; 8.0) mmHg; *p* = 0.038].

**Conclusion:**

Despite a lot of information regarding cardiac impairment due to SARS-CoV2, our study does not suggest an increased risk for developing clinically significant heart changes during the 1-year follow-up. Based on our results, routine echocardiography and biomarkers collection is currently not recommended after COVID-19 recovery.

## Introduction

The beginning of 2020 witnessed the COVID-19 pandemic caused by the novel coronavirus designated as SARS-CoV-2 sweep around the world. Its influence on the global population, health care systems, global economy, and our daily lives has been unprecedented. There is no doubt that the coronavirus pandemic has had an influence on the health and well-being of the population.

COVID-19 is primarily a respiratory disease with potential extra pulmonary manifestations. The involvement of the heart, kidneys, gastrointestinal tract and brain has been described during the acute phase. Its lethality is generally low, with Covid-related deaths affecting mainly the population of patients older than 60 and people with serious underlying chronic diseases. Most patients recover within 1 or 2 weeks. However, the virus’s impact on the population is not only about the number of victims. A non-negligible proportion of patients (approx. 5–10%) fail to recuperate fully. The condition, where the symptoms persist beyond week 4 or beyond week 12, are called “long COVID” and “post-Covid syndrome,” respectively (1).

The proportion of patients with persisting symptomatology is only about 5% approximately, but with the number of people infected worldwide, dealing with post-Covid patients can be challenging for health care systems. These symptoms include fatigue, cough, headache, shortness of breath, chest pains, joint pain, brain fog, gastrointestinal issues, skin changes, loss of taste and smell, along with neuropsychiatric symptoms such as insomnia, anxiety, depression, and delirium (2). These problems may develop regardless of the severity of acute infection. The management of these long haulers is nowadays based especially on the effort to rule out other common causes of the symptomatology present (3), cardiovascular pathology included. When the manifestation of post-Covid syndrome is highly varied and different phenotypes have been recently established (4), an interdisciplinary approach is recommended and physicians across many fields of medicine are involved. This investigation is consuming in terms of time and personnel, is expensive, and the efficacy is not very high. There is thus a need for a comprehensive evidence-based plan to manage post-Covid symptoms. No specific therapy is available so far and recommendations in cardiology are mostly guided by our experiences form non-COVID diseases (5).

Many symptoms of post-Covid syndrome may overlap with those of a cardiovascular origin. Despite data from MRI studies about myocardial damage (6), the clinical significance of these findings is unknown. Large epidemiologic studies provided data about higher cardiovascular risk after hospitalization for COVID-19 (7), but evidence-based recommendation for single patient, even with ongoing symptomatology, is still lacking.

To better understand the cardiovascular sequelae of COVID-19, we initiated a prospective observational cohort study called CoSuBr (COvid SUrvivals in BRno). The aim of our study was to help the physicians and their patients with the tools commonly available in their practise–echocardiography and biomarkers–to select the individuals for more extensive investigation.

## Materials and methods

We recruited patients in outpatient clinic for postCOVID care at least 6 weeks after their recovery from acute symptomatic COVID-19 infection (PCR verified SARS-CoV-2 from a nasopharyngeal swab). After signing an informed consent form, a clinician interviewed all patients. The demography (age, sex), medical history and data about the acute phase of COVID-19 infection was collected [severity of the disease (mild without dyspnea or abnormal chest imaging, moderate for evidence of lower respiratory tract infection without decrease of oxygen saturation < 94%; severe with the necessity of oxygen; critical with respiratory failure and/or shock), need for oxygen/artificial ventilation, intensive care, type and duration of the symptomatology, treatment]. Special interest was paid to ongoing ailments. The follow-up was scheduled at the beginning (Visit 1), after 3 months (Visit 2), and then after 12 months. All of the patients underwent transthoracic echocardiography (Visits 1, 2, and 3). Venous blood samples were collected from all participants for complete blood count, serum creatinine, liver tests, hemoglobin, NT-proBNP, troponin, lipid spectrum (V1, 2, 3). Patients with ongoing respiratory symptomatology underwent chest imaging (X-ray or lung CT).

The trial protocol was approved by the institutional review board at University Hospital Brno (08-100620/EK). The data were managed using REDCap electronic data capture tools, and a data analysis was carried out using SAS software. Mean (and Standard Deviation) is used to present average values for normal data distribution, and median (and interquartile range) is used to present data of non-normal distribution. Prevalence is reported as the number of and percentage of patients reporting the symptom within the group. A paired *t*-test or its non-parametric alternative (Wilcoxon test) was used to compare variables for one subject between the visits.

## Results

58.5% of the study group (*n* = 106) were female, while the mean age was 45.7 years (range 18–77 years). The most prevalent comorbidities were arterial hypertension (*n* = 21, 20.2%); others included obesity, e.g., BMI over 30 (*n* = 12, 11.5%), hepatic steatosis (*n* = 7, 6.7%), asthma, and renal insufficiency (both *n* = 6, 5.8%), Table 1.

**TABLE 1:** Demographic and baseline characteristics.

Parameter	Statistics	*n* = 106
Female	n (%)	62 (58.5)
Age (years)	Mean (SD)	45.7 (14.48)
	Q1/Median/Q3	34.0/46.5/57.0
	Min/Max	18/77
BMI (kg/m2)	Mean (SD)	26.6 (4.81)
	Q1/Median/Q3	23.0/26.0/29.4
	Min/Max	19/43
**Comorbidities**		
Hypertension	n (%)	21 (20.2)
Obesity	n (%)	12 (11.5)
Asthma bronchiale	n (%)	6 (5.8)
Renal insufficiency	n (%)	6 (5.8)
Depression	n (%)	3 (2.9)
Thromboembolic disease	n (%)	2 (1.9)

SD, standard deviation; Q1 = 25% quartile; Q3 = 75% quartile.

The mean time interval between the onset of the infection and the follow-up visit was 107 days. One quarter of the patients required hospitalization during the acute phase of the disease, while the rest recovered at home. The majority (74.4%) of patients suffered a mild form of the disease, while 4.7, 17.9, and 2.8% suffered moderate, severe, or critical forms, respectively.

At the time of the first evaluation, 63.5% of the patients reported persistent symptoms, thus fulfilling the criteria of post-Covid syndrome, while more than half of the whole group mentioned at least one symptom of possible cardiac origin (breathing problems, palpitations, exercise intolerance, fatigue).

During the 1-year follow-up, one patient was diagnosed with paroxysmal atrial fibrillation, and one with second degree atrioventricular block; in one subject coronary artery disease was diagnosed. In three patients mild pericardial effusions were found with no intervention necessary. No left or right ventricle dysfunction was found on echocardiography. No patient died during follow-up.

**FIGURE 1:**
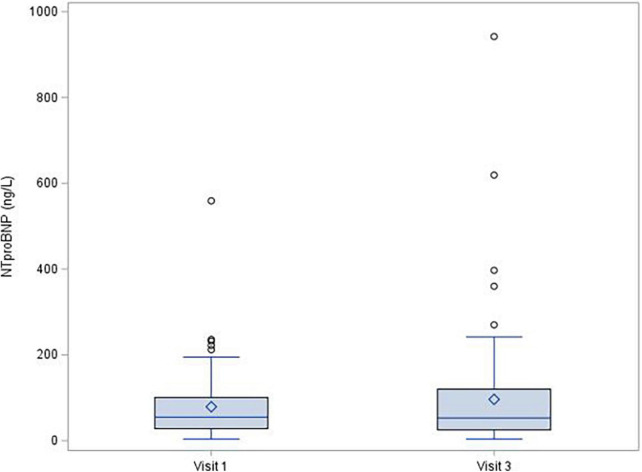
NT-proBNP, change between Visit 1 a Visit 3, *p* = 0.315.

**FIGURE 2:**
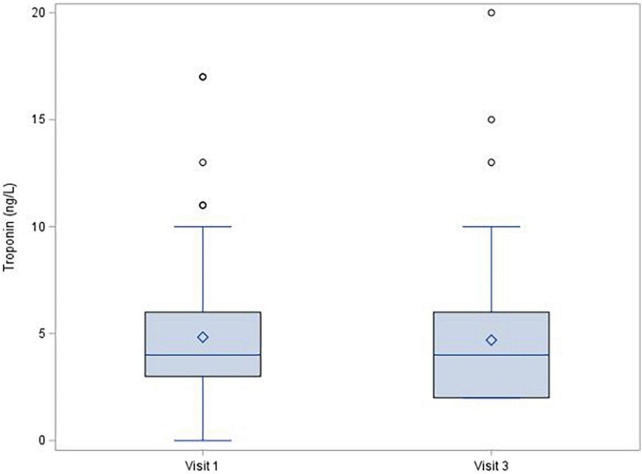
Troponin level, change between Visit 1 a Visit 3, *p* = 0.380.

**FIGURE 3:**
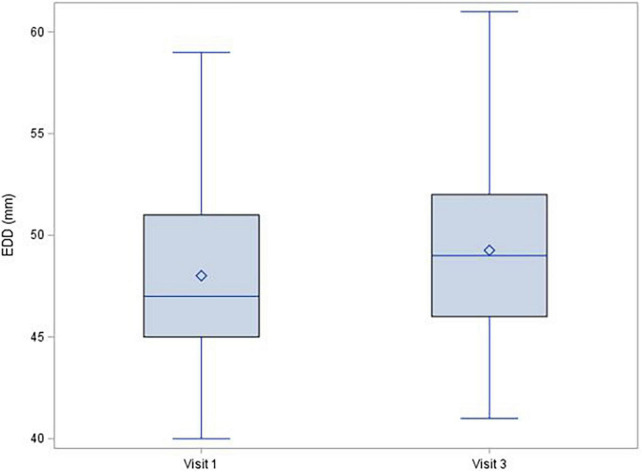
Left ventricle end diastolic diameter, change between Visit 1 a Visit 3, *p* = 0.001.

During the 1-year follow-up after COVID-19 infection, left ventricle ejection fraction showed no significant decrease [median (IQR) change was −1.0 (−6.0; 4.0)%, *p* = 0.150], and there were no changes of troponin (mean change −0.1 ± 1.72 ng/L; *p* = 0.380; Figure 1) or NT-proBNP [median (IQR) change 2.0 (−20.0; 29.0) pg/mL; *p* = 0.315; Figure 2]. There was a mild decrease in the right ventricle end diastolic diameter (-mean change 2.3 ± 5.61 mm, *p* < 0.001), while no right ventricle dysfunction was detected. There was very mild progress in left ventricle diastolic diameter [median (IQR) change 1.0 (−1.0; 4.0) mm; *p* = 0.001; Figure 3] between V1 and V3, mild enlargement of the left atrium (mean change 1.2 ± 4.17 mm; *p* = 0.021) and non-significant trend to impairment of left ventricle diastolic dysfunction. There was a mild change in pulmonary artery systolic pressure [median (IQR) change 3.0 (−2.0; 8.0) mmHg; *p* = 0.038].

Changes in echocardiographic parameters and biomarkers are summarized in Table 2.

**TABLE 2:** Change in echocardiographic parameters and biomarkers during 1 year follow-up.

Parameters	Statistics	Visit 1 (baseline)	Visit 3 (12-month follow-up)	Change from baseline at Visit 3	*p*-value*
NTproBNP (ng/L)	Mean ± SD	79.1 ± 80.60	96.4 ± 137.80	17.4 ± 94.39	0.315*
	Median (Q1; Q3)	55.0 (28.0; 101.0)	53.0 (25.0; 120.0)	2.0 (−20.0; 29.0)	
Troponin (ng/L)	Mean ± SD	4.8 ± 3.26	4.7 ± 3.31	−0.1 ± 1.72	0.380
	Median (Q1; Q3)	4.0 (3.0; 6.0)	4.0 (2.0; 6.0)	0.0 (−1.0; 1.0)	
EDD (mm)	Mean ± SD	48.0 ± 4.40	49.3 ± 4.21	1.2 ± 3.41	0.001
	Median (Q1; Q3)	47.0 (45.0; 51.0)	49.0 (46.0; 52.0)	1.0 (−1.0; 4.0)	
LV EF (%)	Mean ± SD	62.1 ± 5.33	60.8 ± 6.24	−1.3 ± 7.88	0.150
	Median (Q1; Q3)	60.0 (60.0; 65.0)	61.0 (55.0; 66.0)	−1.0 (−6.0; 4.0)	
LA (mm)	Mean ± SD	37.1 ± 5.32	38.2 ± 5.65	1.2 ± 4.17	0.021*
	Median (Q1; Q3)	37.0 (34.0; 40.0)	38.0 (34.0; 41.0)	1.0 (−1.0; 3.0)	
PASP (mmHg)	Mean ± SD	24.8 ± 8.93	27.5 ± 6.83	2.7 ± 7.99	0.038
	Median (Q1; Q3)	25.0 (19.0; 31.0)	28.0 (25.0; 31.0)	3.0 (−2.0; 8.0)	
PK (mm)	Mean ± SD	29.1 ± 5.53	26.8 ± 3.83	−2.3 ± 5.61	< 0.001*
	Median (Q1; Q3)	28.0 (25.0; 33.0)	27.0 (24.0; 29.0)	−1.0 (−6.0; 1.0)	

n, number of values available for paired comparison; SD, standard deviation; Q1 = 25% quartile; Q3 = 75% quartile. *p-value of non-parametric Wilcoxon test (for parameters with violated normality).

## Discussion

The impairment of the cardiovascular system during the acute phase of COVID-19 infection has been well-described (8), as is the importance of bedside echocardiography for decision-making in those with severe or critical forms of the infection (9). According to on-line survey performed by the European Association of Cardiovascular Imaging (EACVI) during the acute course of the disease, one third of echocardiographic studies performed in Covid-positive patients led to change management (6). The impact of hypoxia and cytokine storm during severe inflammatory responses in predisposed individuals can cause a broad spectrum of cardiovascular pathologies. In addition, myocardial damage caused by SARS-CoV-2 is suspected for reasons including its spike affinity to the ACE2 receptor binding domain, which plays essential role in renin-angiotensin system (10).

Even though the COVID-19 pandemic has been ongoing for more than 2 years, there are only several studies about the prevalence of myocardial injury and cardiac outcome in individual post-Covid patients, especially in those with the mild course of the disease. The very first data from spring 2020, from magnetic resonance heart studies, suggested almost 60% of patients with ongoing inflammation after COVID-19, when the myocardial damage was not dependent on the severity of acute infection (6). Given the proportion of the population having been infected during the several months following the publication of that study, it seemed that the coronavirus pandemic would be followed by a pandemic of heart failures. Based on these data, we initiated a study with a longer follow-up. Also a research group from China published the results of their work (27 pt involved), which showed a normal echocardiogram in all patients 6 months after COVID infection; however, according to magnetic resonance there was persistent cardiac involvement in almost 30% of the study subjects (11). Negative echocardiographic findings with a short-term follow-up were also published by Italian researchers (12). In addition, a team from Austria described a significant improvement in symptoms and cardiopulmonary status during 100 days of observation after acute COVID-19 despite a significant proportion of patients with persisting symptomatology and CT changes (13). These findings are in concordance with our own results from our 1-year follow-up. The data showing subclinical changes from mild alterations of the global longitudinal strain of the left ventricle is interesting (14) but such findings are not necessarily of clinical importance. The very mild trend to left ventricle diastolic impairment and to left atrium enlargement which we observed in our study during follow-up may be the first possible sign of some SARS-CoV-2-induced heart changes, but despite the statistical significance of these findings, these very mild changes are not of clinical importance.

Data analyses from national healthcare databases from the US Department of Veterans Affairs show very strong evidence of cardiovascular risk for the post-Covid population. Investigators compared a large control cohort (*n* = 5,637,647) and 153,760 individuals with COVID-19 and show a substantial increase in cardiovascular disease, including cerebrovascular disorders (hazard ratio 1.53), dysrhythmias (HR 1.69), ischemic (HR 1.66), and non-ischemic heart disease, pericarditis, myocarditis, heart failure (HR 1.72) and thromboembolic disease (HR 2.39) between 30 days and 1 year after acute illness (15). The risk was slightly higher even in the non-hospitalized, further increased with the severity of acute disease, and the difference was strongest in those who needed intensive care. Other study done by Wang et al. also provided data for increased cardiovascular risk during 1 year follow up after positive test for COVID-19 compared to population with negative one: hazard ratio for any cardiovascular outcome was 1.552 [1.526–1.578] and 4.406 [2.890–6.716] for myocarditis (7). This is important especially for public health care systems. When the infections went through the population in several waves and a large proportion was infected, a substantial increase in health care usage can be estimated during the subsequent months and years.

Possible explanation for increased rate of cardiovascular endpoints after hospitalization for COVID-19 can be hidden in the spectrum of patients, who need to be admitted to the hospital for acute infection. The incidence of cardiovascular complications during the acute phase is much higher in the elderly and especially in those admitted to intensive care and that requiring organ support. In older people and those with a number of comorbidities, late cardiovascular complications are more common. It is complicated to differentiate the effects of the virus itself, the impact of other diseases, aging, and even the harm caused by intensive care and artificial ventilation. The necessity of hospitalization for acute viral infection can be only the marker of increased cardiovascular risk in the future.

Considering direct viral damage to the heart a systematic review of thirty-five very heterogenic studies evaluating cardiac sequelae in 52,609 patients also suggest some subclinical changes in the early phase of recovery and later development of diastolic dysfunction (16). These results are based on magnetic resonance imaging, echocardiography or biomarkers collection.

Even if our data set is much smaller, our longer follow-up with serial measurements and higher proportion of patients with ongoing symptomatology do not support these findings. More than half of the study group (63.5%) fulfilled the criteria of post-Covid syndrome, which is given by the source of our subjects, which was mainly outpatient clinic for post-Covid care. A majority of our patients had a mild course of the acute disease, which is the same proportion of COVID-19 disease severity globally. Our population represents a wide spectrum of ages.

Except for hypertension (20%), none of our patient suffers from cardiovascular disease before the enrolment. Rate of repeated infection was rare–only four patients had recidivism of COVID during follow up. Also the analyses of vaccinated and unvaccinated is impossible, because the recruitment to the study was almost done, when the vaccina become available.

Despite the high rate of symptomatic patients in our study group the cardiovascular clinical endpoints were quite rare and their distribution was in the oldest tenth of our study group. One patient was diagnosed with *de novo* paroxysmal atrial fibrillation (female, 73 years old, dilated left atrium), another with atrioventricular block with an indication for implantation of a pacemaker (male, 71 years old; cardiac MRI did not find any signs of myocardial inflammation); one subject (male, 69 years old) was diagnosed on Visit 2 with coronary artery disease due to atherosclerosis and required revascularization by percutaneous coronary intervention. All these three patients had NT-proBNP and high-sensitive troponin in a highest quartile, but the low incidence of endpoints is insufficient for statistical analyses. None of these findings are, in our opinion, suspected to be the direct result of SARS-CoV-2 infection. One female was prescribed a beta-blocker for inadequate sinus tachycardia and palpitations. In three patients, mild pericardial effusion was found with no intervention necessary. No patients died during follow-up.

Acute COVID-19 is associated with right ventricle failure and pulmonary hypertension in 39% of patients (17). This is not only due to an increase in pulmonary vascular resistance during lung inflammation but also owing to SARS-CoV2-associated thromboembolic disease. Despite this, we did not note any right ventricle failure in our group of post-Covid patients and the incidence of pulmonary hypertension is very rare, with a mild trend toward a decrease in pulmonary artery pressure during the 1-year follow-up.

## Conclusion

Despite a very high proportion of patients with ongoing symptoms of possible cardiac origin (such as shortness of breath, loss of exercise capacity and chest pain), using commonly available methods proven cardiac impairment was rare in our study. Based on our results, routine echocardiography and biomarkers collection is currently not recommended after COVID-19 recovery.

## Data availability statement

The raw data supporting the conclusions of this article will be made available by the authors, without undue reservation.

## Ethics statement

The studies involving human participants were reviewed and approved by Ethic Comitee FN Brno, 08-100620/EK. The patients/participants provided their written informed consent to participate in this study.

## Author contributions

GM, MR, and EB organized the study, prepared the study protocol, and wrote the first draft. GM and JH organized the database. RS performed the statistical analysis. All authors contributed to data evaluation, manuscript revision, read, and approved the submitted version.
